# Health Tract, No. 2

**Published:** 1860-02

**Authors:** 


### HEALTH TRACT, No. 2.

### SUMMER SOURS.

Physiological research has fully established the fact that acids promote the separation of the bile from the blood, which is then passed from the system, thus preventing fevers, the prevailing diseases of summer. All fevers are "bilious," that is, the bile is in the blood. Whatever is antagonistic of fever is cooling. It is a common saying that fruits are "cooling," and also berries of every description; it is because the acidity which they contain aids in separating the bile from the blood, that is, aids in purifying the blood. Hence the great yearning for greens and lettuce and salads in the early spring, these being eaten with vinegar; hence also the taste for something sour, for lemonades, on an attack of fever.

But this being the case, it is easy to see, that we nullify the good effects of fruits and berries, in proportion as we eat them with sugar, or even sweet milk or cream. If we eat them in their natural state, fresh, ripe, perfect, it is almost impossible to eat too many, to eat enough to hurt us, especially if we eat them alone, not taking any liquid with them whatever. Hence also is buttermilk, or even common sour milk promotive of health in summer time. Sweet milk tends to biliousness in sedentary people; sour milk is antagonistic. The Greeks and Turks are passionately fond of sour milk. The shepherds use rennet, and the milk-dealers alum to make it sour the sooner. Buttermilk acts like watermelons on the system.

### THE DIFFERENCE.

When a simpleton wants to get well, he buys something "to take;" a philosopher gets something "to do;" and it is owing to the circumstance, that the latter has been in a minority almost undistinguishable in all nations and ages, that doctors are princes, instead of paupers; live like gentlemen, instead of cracking rocks for the turnpike.

### POISONOUS BITES.

During the increased travel of summer, the bites from insects and reptiles of various kinds are of frequent occurrence. Persons of healthful blood are bitten with impunity sometimes, while those in feeble health suffer distressing, and sometimes fatal, consequences.

Almost all poisonous bites arise from the acidity of the virus; it then follows that an alkali is the best antidote, because an alkali and an acid are as much opposed to each other as light and darkness, as sweet and sour. And as expedition is sometimes the life of a man, it is of considerable practical importance to know what is the most universally available remedy. A handful of the fresh ashes of wood is the most generally accessible; pour on enough water, hot is best, to cover it, stir it quickly, and either apply the fluid part, that is the ley, with a rag or sponge, or have less water, and apply a poultice made of simple water and fresh wood-ashes. Renew the poultice every half-hour until the hurting is entirely removed. As to minor insects, the relief is almost instantaneous. The next most convenient remedy is common spirits of hartshorn, a small vial of which should be in every family, and in every traveller's trunk or carpet-bag, in summer-time at least. Saleratus, dampened and applied to the wound or stung place, is not as powerful as hartshorn. It failed recently to cure the sting of a bee, the gentleman dying in convulsions within an hour after he was stung; this arose from some peculiarity of constitution, an "Idiosyncracy," as physicians term it.

